# The relation between autism and psychosis: overlapping and differing features

**DOI:** 10.1192/j.eurpsy.2024.955

**Published:** 2024-08-27

**Authors:** M. B. Ruas Resende, F. Agostinho, R. Nogueira, F. A. Silva, R. Lousada, D. Cotovio

**Affiliations:** Psychiatry, Hospital de Loures, Lisbon, Portugal

## Abstract

**Introduction:**

Autism spectrum disorders (ASD) and schizophrenia (SCZ) have a strong historic connection. At the beginning of the 20th century when referring to schizophrenic patients Eugen Bleuler used the term autism to describe the apparent withdrawal from the outside world. Other authors also emphasized the association between this two entities. In fact, only in DSM-III were these disorders placed in different diagnostic categories. Today, even though this nosological vision still prevails, a growing number of studies have shown significant overlaps between the two disorders. Patients with the diagnosis of ASD often experience psychotic symptoms and similarly schizophrenic patients have a high prevalence of autistic traits.

**Objectives:**

To clarify the distinction between ASD and psychotic disorders, namely to help the clinical and phenomenological distinction between patients with a primary psychotic disorder versus patients with the diagnosis of an autism spectrum disorder that might also experience psychotic symptoms.

**Methods:**

Research on UpToDate using the terms “Autism Spectrum Disorders”; “Schizophrenia” and “psychosis”.

**Results:**

Delusional beliefs and paranoid ideation are common findings in autistic individuals in the same way that they constitute one of the main features of schizophrenia spectrum disorders. However, in ASD individuals one must be vigilant of its distinction with “childish fantasies”. Both disorders (ASD and SCZ spectrum disorders) share Theory of Mind (ToM) impairments that contribute to the development of paranoia.

Sensory anomalies are common in ASD and might be confused with hallucinations. However, anomalous perceptual experiences can and do often happen in ASD and are clinically overlapping with hallucinatory phenomena. In the case of a neurodevelopment disorder, however, they could probably be better understood as a part of it more than the signal of a co-ocurring psychotic disorder. Attenuated psychotic symptoms pose an even more complex subject because of the overlap between autistic symptoms and subclinical psychotic symptoms. Another area that poses diagnostic difficulties has to do with the distinction between negative symptoms seen in schizophrenia and autistic symptoms. Lack of emotional reciprocity in ASD can be confused with “blunted” affect in schizophrenia.

Other overlapping features between these two entities can be identified.

**Conclusions:**

The diagnostic boundaries between ASD and SCZ are not always clear. Their overlapping characteristics and potential co-occurrence might pose important diagnostic challenges in clinical practice. The clinical course of both diseases frequently represents a key element for the differential diagnosis between autism and psychosis. The profound knowledge of these two entities is of extreme importance contributing to the implementation of more targeted and effective management strategies.

**Disclosure of Interest:**

None Declared

